# Structural Dynamics of the GW182 Silencing Domain Including its RNA Recognition motif (RRM) Revealed by Hydrogen-Deuterium Exchange Mass Spectrometry

**DOI:** 10.1007/s13361-017-1830-9

**Published:** 2017-10-27

**Authors:** Maja K. Cieplak-Rotowska, Krzysztof Tarnowski, Marcin Rubin, Marc R. Fabian, Nahum Sonenberg, Michal Dadlez, Anna Niedzwiecka

**Affiliations:** 1 Division of Biophysics, Institute of Experimental Physics, Faculty of Physics, University of Warsaw, 02-089 Warsaw, Poland; 2 Laboratory of Mass Spectrometry, Institute of Biochemistry and Biophysics, Polish Academy of Sciences, PL-02106 Warsaw, Poland; 3 Lady Davis Institute for Medical Research, Jewish General Hospital, Montréal, Québec Canada; 4 Department of Oncology, McGill University, Montréal, Québec Canada; 5 Department of Biochemistry, McGill University, Montréal, Québec Canada; 6 Goodman Cancer Center, McGill University, Montréal, Québec Canada; 7 Laboratory of Biological Physics, Institute of Physics, Polish Academy of Sciences, Aleja Lotnikow 32/46, PL-02668 Warsaw, Poland

**Keywords:** Hydrogen–deuterium exchange, Mass spectrometry, Structural dynamics, Intrinsically disordered proteins, GW182, RNA recognition motif (RRM)

## Abstract

The human GW182 protein plays an essential role in micro(mi)RNA-dependent gene silencing. miRNA silencing is mediated, in part, by a GW182 C-terminal region called the silencing domain, which interacts with the poly(A) binding protein and the CCR4-NOT deadenylase complex to repress protein synthesis. Structural studies of this GW182 fragment are challenging due to its predicted intrinsically disordered character, except for its RRM domain. However, detailed insights into the properties of proteins containing disordered regions can be provided by hydrogen–deuterium exchange mass spectrometry (HDX/MS). In this work, we applied HDX/MS to define the structural state of the GW182 silencing domain. HDX/MS analysis revealed that this domain is clearly divided into a natively unstructured part, including the CCR4-NOT interacting motif 1, and a distinct RRM domain. The GW182 RRM has a very dynamic structure, since water molecules can penetrate the whole domain in 2 h. The finding of this high structural dynamics sheds new light on the RRM structure. Though this domain is one of the most frequently occurring canonical protein domains in eukaryotes, these results are – to our knowledge – the first HDX/MS characteristics of an RRM. The HDX/MS studies show also that the α2 helix of the RRM can display EX1 behavior after a freezing-thawing cycle. This means that the RRM structure is sensitive to environmental conditions and can change its conformation, which suggests that the state of the RRM containing proteins should be checked by HDX/MS in regard of the conformational uniformity.

**Electronic supplementary material:**

The online version of this article (10.1007/s13361-017-1830-9) contains supplementary material, which is available to authorized users.

**Figure:**
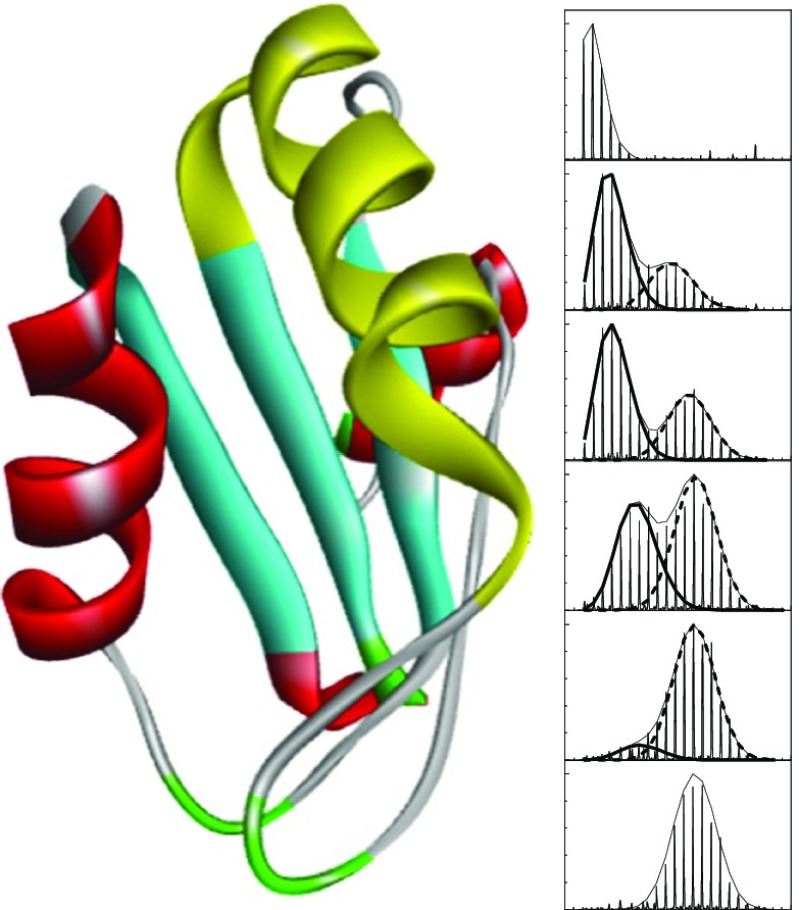
Graphical Abstract

## Introduction

In recent years, there have appeared a significant number of works proving that structural dynamics governs the function of biological molecules to no less extent than the protein sequence and its 3D structure [1–3]. Many biologically important issues related to conformational changes were addressed by an approach that combined hydrogen–deuterium exchange with mass spectrometry [4–7].

**Figure 1:**
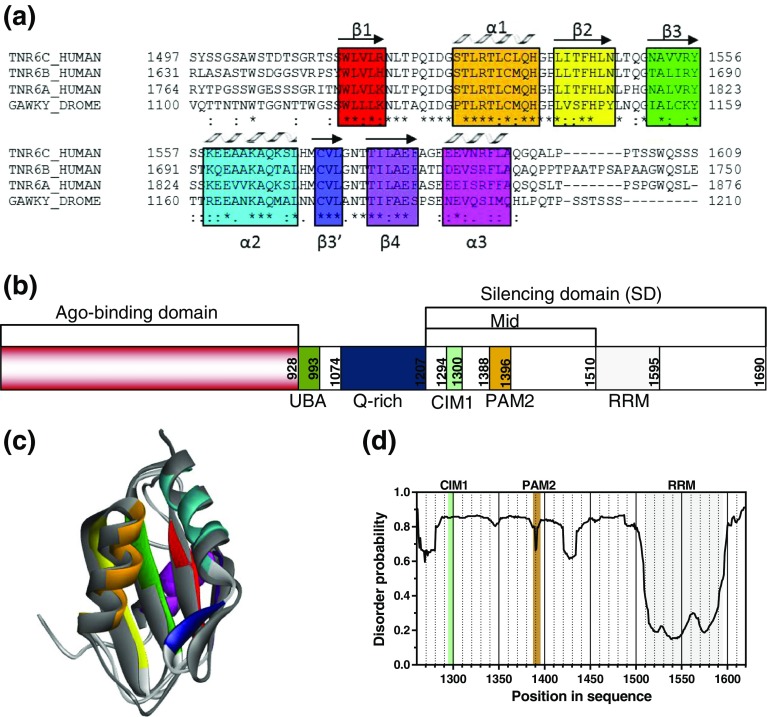
**(a)** Sequence alignments of the RRM domains of different GW182 isoforms from human, mouse, and *Drosophila melanogaster*. Secondary structural elements are marked as color blocks corresponding to the α-helices and β-sheets shown in **(c)**; **(b)** schematic representation of human GW182 (TNRC6C): an Ago-binding domain, a ubiquitin associated domain (UBA), a glutamine-rich (Q-rich) region, and the silencing domain (SD). SD contains a CCR4-NOT interacting motif (CIM1) [11], a PABP-interacting motif (PAM2), and RRM; **(c)** predicted human GW182 RRM structure shown as a ribbon colored according to the secondary structural elements shown in Figure **(a)**, aligned with the NMR *D. melanogaster* RRM structure (grey) [12]; **(d)** disorder tendency of the human GW182 silencing domain (SD; residues 1260-1620); CIM1 is marked in mint, PAM2 in dark gold, RRM in light grey

The RRM domain is thought to be one of the most abundant globular protein domains in eukaryotes [8]. To our surprise, among over 115,000 protein structures deposited in the RCSB PDB, only about 340 structures of RRM domains can be found. Among them, more than 200 were resolved by NMR and about 140 by X-ray crystallography, and almost 50% of the RRM structures became known only during the last 5 y. This brief statistic reflects the dynamic structural character of this protein domain that can lead to difficulties in crystallization of these proteins. A canonical RNA recognition motif (RRM) is defined by two conserved ribonucleoprotein motifs (RNPs) within an ~80 amino acid long fold consisting of a four-stranded antiparallel β-sheet supported by two α-helices, arranged in the order of β1α1β2β3α2β4 [8–10] (Figure 1a). Usually, the β3 (RNP1) and β1 (RNP2) strands contain conserved aromatic residues that make direct contact to the RNA chain. Interesting exceptions among RRMs are related to the mRNA 5′ cap interacting proteins, i.e., the nuclear cap-binding complex (CBC) [13] and poly(A)-specific ribonuclease (PARN) [14]. The CBC RRM called CBP20 binds the 5’ terminus of the polymerase II transcripts chain via Tyr43 residue located at β1 in the opposite direction than other RNA-interacting RRMs [15–17], whereas the PARN RRM binds to the mRNA 5′ cap via Trp475 located entirely at its outer surface [18–20].

The GW182 protein is one of the crucial players in miRNA-mediated gene silencing, since it specifically interacts with the Argonaute (Ago) proteins [21–23] and recruits the multi-subunit CCR4-NOT deadenylase complex [11, 24, 25] to the targeted mRNA to trigger both translational repression at the 5′ terminus and removal of the mRNA 3′ poly(A) tail [26–28]. The first human GW182 protein discovered, now known also as TNRC6A (trinucleotide repeat-containing gene 6 protein A), has many glycine-tryptophan (GW) repeats and a predicted mass of 182 kDa [29]. It was discovered in human cells as the antigen recognized by an autoimmune serum from a patient suffering from motor and sensory polyneuropathy [30]. There are usually three paralogs of GW182 in vertebrates: TNRC6A, TNRC6B, and TNRC6C. Invertebrates usually possess only one protein, e.g*.*, *Drosophila melanogaster* GW182 (also known as Gawky), but three paralogs were found in mosquitos [30, 31]. In a recent phylogenetic study [31], it was found that GW182 proteins are animal specific and a single copy of the gene arose with the emergence of multicellularity. Then three copies of the gene appeared in the last common ancestor of vertebrates as a result of two rounds of whole genome duplication events [31]. TNRC6C is thought to be the founding member of the vertebrate family of GW182 proteins and is the ortholog of the invertebrate GW182 [31]. The three human paralogs are considered to be redundant [32] on the basis that these proteins associate with all four human Ago proteins and a common set of miRNA targets [23, 26, 33], and silencing is efficiently suppressed when at least two of the proteins are co-depleted [34].

GW182s are predicted to be mostly disordered except for two globular domains: the ubiquitin associated domain (UBA) and the RRM (Figure 1b) [30]. The structure of the *D. melanogaster* GW182 RRM domain (residues 1510-1595 in TNRC6C) was solved by NMR [12] and revealed the presence of an additional α3-helix on the side of the β-sheet that is solvent-exposed in canonical RRMs (Figure 1a, c) [8]. The α3-helix is tightly bound to the β-sheet, making it inaccessible to the solvent and ligands. In consequence, the *Dm* GW182 RRM does not bind RNA in vitro [12]*.* The GW182 RRMs have a high sequence homology (Figure 1a), which suggests that the human RRMs are also incapable of RNA binding. It was proposed that the GW182 RRM might be involved instead in protein–protein interactions using the opposite side of the domain, where α1 and α2 helices together with β3′ and β4 strands form a broader hydrophobic cleft [12], which could potentially be utilized for such interactions, thus contributing to gene silencing. Contrary to these expectations, no evidence of such direct interactions was found, even though the presence of the RRM domain in the GW182 sequence was shown to be required for efficient gene silencing [12, 32]. This observation corresponds better to another putative role that could be fulfilled by RRM domains, i.e., stabilization of a protein structure that enables assembling of a required functional bipartite motif composed of unstructured regions flanking the folded RRM, similarly as, e.g., in case of a bimodular non-classical nuclear localization signal of ADAR1 enzyme [35]. This role is particularly plausible for the RRM belonging to GW182, since four of the remaining regions of the protein, i.e., a glutamine-rich (Q-rich) domain and three GW-rich regions: an N-terminal Argonaute proteins-binding domain, a middle domain (Mid) between Q-rich and RRM, and a C-terminal part [29, 30], are supposed to be disordered (Figure 1b). The N-terminal GW repeats mediate the interaction with Ago through tryptophans [22, 28, 36]. The Q-rich region (residues 1074-1207 in TNRC6C) was implicated to play a role in P-body localization [28, 30]. The Mid region has a highly conserved PAM2 motif that interacts specifically with the cytoplasmic poly(A)-binding protein (PABPC1) [37, 38].

miRNA-dependent gene silencing by the GW182 protein is mediated by its C-terminal half called the silencing domain (SD), which – when artificially tethered – can trigger silencing of reporters bypassing the involvement of miRNAs and Argonauts [26, 33, 39]. The silencing activity of the GW182 SD stems from its ability to bind with deadenylase complexes: CCR4-NOT and Pan2-Pan3, and also with PABP [11, 24, 25, 37, 38]. The interaction of the human GW182 with the CCR4-NOT components is thought to be mediated by the Mid and C-terminal unstructured regions of the SD, among them a region encompassing residues 1286-1306 in TNRC6C, termed the CCR4-NOT interacting motif 1 (CIM1, Figure 1b) [11, 24]. Binding of the Pan2–Pan3 complex requires the interaction of the N- and C-term regions surrounding the RRM [24, 25]. Other GW182 properties were reviewed in more detail by Braun et al. [32].

Structural studies of the GW182 silencing domain is challenging because of its intrinsically disordered character. There is a continuum of protein structures ranging in the degree of disorder from close to none in globular proteins through proteins with ordered domains linked via disordered linkers, molten globules to completely unstructured proteins (intrinsically disordered proteins, IDPs; reviewed in [40]). This is linked to a broad range of observed levels of protein structural dynamics. It was shown that the frequency of breaking of main chain H-bonds, which reflects the frequency of deviations of the chain position from the average conformation, may differ by 5–7 orders of magnitude [41]. What we call “protein structure” is just a small window in this spectrum, easy to visualize because of fixed atom positions, whereas the rest of the spectrum of protein states is underappreciated, being more difficult to represent. This phenomenon has been called “a dark-matter in biology” [42].

IDPs have been found to be widespread [43], and involved in such diverse processes as transduction of signals, transport through nuclear pores, regulation of transcription, translation, and the cell cycle, as well as binding of RNA, DNA, and other small ligands [44]. Many, but not all, IDPs gain structure upon binding a partner [45]. IDPs interact with their binding partners differently than globular proteins do. They are able to interact with different target proteins using the same region, which results in adapting different conformations [46]. These proteins use hydrophobic or aromatic residues to mediate interactions more frequently than ordered proteins do [45, 47]. Such protein structures are neither suitable for X-ray crystallography nor for NMR due to their dynamic character or limited solubility. Moreover, they are still challenging even for a computational approach [48].

However, quite detailed insight into the properties of proteins containing disordered regions can be provided by hydrogen–deuterium exchange mass spectrometry (HDX MS) [5]. This technique allows mapping of the stability of H-bonding networks, since the kinetics of the H/D exchange reflects the local dynamics of the protein structural elements [49–51]. In this work, we reveal the structural dynamics of the human GW182 silencing domain. We provide the experimental evidence that the GW182 SD is completely unstructured except the RRM, which has also a very dynamic structure. These results are – to our knowledge – the first hydrogen–deuterium exchange mass spectrometry ‘fingerprint’ of an RRM domain.

## Materials and Methods

### Protein Expression and Purification

Experiments were conducted on the GW182 fragment denoted as the silencing domain (SD, formerly SD10 in [11]), encompassing residues 1260-1620 of the TNRC6C paralog (isoform 1, UniProt Q9HCJ0), with a predicted mass of 39819.9 Da, and confirmed mass of 39822 ± 5 Da. SD was cloned into pET28b(+) His-Sumo vector using *EcoRI* and *XhoI* restriction sites and purified on a His-trap column (GE Healthcare) on AKTA FPLC, followed by in-solution cleavage by CoolCutter (Genecopoeia), and purified again on a His-trap column. Before the experiments, the protein was dialyzed in 50 mM Tris, 150 mM NaCl, 2 mM DTT, 1 mM EDTA, pH 7.0, and concentrated using Amicon Ultra Centrifugal Filters (Merck Millipore) at 4 °C. In most experiments, freshly prepared protein was used. The frozen fraction of the protein, for which the double conformation was found, was stored at –80 °C with 10% glycerol.

Human full length eIF4E was expressed and purified as described previously for the murine homolog [52].

### Hydrogen–Deuterium Exchange Experiments

Each set of experiments was performed four times, essentially as described previously [52, 53]. Each exchange and control measurement in the set was repeated at least three times. The protein was used at initial concentrations of 60 or 200 μM (nonfrozen samples), and 15 or 20 μM (frozen-thawed samples). A typical set of experiments consisted of mass spectra measurements (LC-MS runs) collected after several time intervals (10 s, 1 min, 20 min, 2 h, and in some cases 24 h) of H/D exchange, triggered by 1:10 dilution in heavy water (99.8%, Cambridge Isotope Laboratories, Inc.)-based buffer (50 mM Tris, 150 mM NaCl, 2 mM DTT, 1 mM EDTA, pD 7.0). The deuterium level in the heavy water-based buffer system after adding the chemicals was 99.27%. pD values were measured on pH-meter (pH 1000 L, pHenomenal) and uncorrected. HDX reaction in a volume of 50 μL sample was quenched by adding 10 μL of stop buffer (2 M glycine, 4 M guanidine hydrochloride, 150 mM NaCl, pD 2.5 in 99.8% D_2_O). Two control measurements were performed [52, 53]. The first control experiment was related to the residual exchange that might still occur after quenching. This process is called “in-exchange”. The control was obtained by adding stop buffer first, then diluting the sample in the heavy water-based buffer, in order to correct for a possible imperfect arrest of the H/D exchange reaction at pD 2.5. Then the residual exchange level was measured identically to the main experiments (see below). The experiments in the second set of control, measuring the maximum level of exchange possible to be detected due to a back D/H exchange during LC-MS, were conducted by in-solution pepsin digestion of protein samples in a mildly denaturing condition, followed by HDX and final MS measurements. For this purpose, the immobilized porcine pepsin (Pierce) was prepared by mixing 50 μL slurry (resin plus supernatant) per one back-exchange sample with a 4-fold excess of wash buffer (200 mM glycine, 0.66 M guanidinium hydrochloride, 150 mM NaCl, pD 2.5 (uncorrected), all in D_2_O), centrifuging at 1000 rcf, removing supernatant, and repeating wash three times. Finally, the resin was mixed with wash buffer in a 1:1 ratio and 50 μL of slurry was used per sample.

Five μL of protein was diluted into 45 μL of deuterated reaction buffer, and the reaction was acidified by adding 10 μL deuterated stop buffer. The solution was incubated with the immobilized pepsin for 4 min at 4 °C with shaking. The sample was centrifuged at maximal speed in a table-top microcentrifuge, and 60 μL of solution was transferred to a fresh Eppendorf tube. The pD value of the sample was raised to ~7 with NaOD. After 2 min of incubation at RT, pD was lowered to ~2.5 by adding DCl. The sample was centrifuged once again at maximal speed to get rid of any beads, and 50 μL was injected into the nanoACQUITY UPLC system (Waters) and analysis continued as for normal exchange samples (see below) except that the pepsin column was taken out.

This enables us to correct directly for the back-exchange of the individual peptides that occurred during the chromatographic separation step performed in the H_2_O milieu. The precise correction for the back-exchange also allowed taking into account the effect of the incomplete exchange in the 90% D_2_O buffer after dilution.

### Mass Spectrometry Measurements

Immediately after HDX quench, samples were passed through an immobilized pepsin column (Poroszyme, Applied Biosystems), placed in HDX Manager system, and kept at 13 °C, with 0.07% formic acid (200 μL/min flow rate) as a mobile phase. Pepsin-generated peptides were then trapped in a C18 trapping column (Acquity BEH C18 VanGuard Pre-Column, Waters), and then separated using liquid chromatography on a reverse-phase column (Acquity UPLC BEH C18 column, Waters) with a 8% to 40% gradient of acetonitrile in 0.1% formic acid at a 40 μL/min flow rate using the nanoAcquity Binary Solvent Manager (Waters). The total run time was 13.5 min. The apparatus (excluding the pepsin column) was kept at 0.5 °C. The carryover problem was excluded since the same columns and elution conditions were applied for both the nonfrozen and frozen-thawed samples, and the latter protein was much less concentrated.

Peptides were then passed for MS analysis into the ion source of SYNAPT G2 HDMS (Waters) (Q-ToF) working in Ion Mobility Mode. The mass spectrometer parameters were set as follows: ESI positive mode, capillary voltage 3 kV, sampling cone 35 V, extraction cone voltage 3 V, source temperature 80 °C, desolvation temperature 175 °C, and desolvation gas flow 800 l/h. Mass spectra were acquired over the *m/z* range of 350 to 1500 in the IMS mode. Ion-mobility separation conditions in TriWave device were as follows: helium cell flow rate 180 mL/min, ion-mobility nitrogen flow rate 90 mL/min, IMS wave velocity 600 m/s, and IMS wave height 40.0 V.

### Numerical Data Analysis

The peptic peptides were identified in nondeuterated buffer based on mass spectra acquired in MSE mode, with the low-energy function voltage of –4 eV and high-energy function voltage of –28 eV, over the *m/z* range of 100 to 1950, in either standard or IMS mode. The isotopic envelopes after exchange were assigned to their corresponding peptide sequence tags using DynamX 2.0 or 3.0 (Waters), based on a peptide list obtained from the triple sequencing analysis of unexchanged sample by ProteinLynx Global Server software (Waters) and a randomized database. The following acceptance criteria were used: minimum intensity threshold of 1000, minimum products per amino acid of 0.25, max MH+ error of 5 ppm. Identification of isotopic envelopes was conducted with retention time tolerance ± 0.3 min, mass tolerance ± 15 ppm, and drift time deviation ± 2 time bins. The acquired data were verified by visual inspection and exported to Excel, R, or Prism 6 (GraphPad) for further analyses. Bimodal isotopic envelopes were exported directly from MassLynx (Waters) and analyzed by HX-Express2 [54].

The fraction of peptide amide hydrogens exchanged to deuterium, *D*_*f*_, for each peptide was calculated as:

1 $$ {D}_f=\frac{M_t-{\overline{M}}_{in}}{{\overline{M}}_{bx}-{\overline{M}}_{in}} $$

where *M*_*t*_ – the average mass of the peptide after a given exchange time, *t*; ${\overline{M}}_{in}$ – the average mass of the peptide in the in-exchange control samples; ${\overline{M}}_{bx}$ – the average mass of the peptide in the back-exchange control samples.

The time dependence of the fraction exchanged for each pepsin generated peptide was analyzed by a nonlinear, least-squares regression assuming for simplicity the existence of a single or dual population of amide protons of a given peptide, that exchange with the kinetics described by either a mono-exponential or bi-exponential equation, respectively:

2 $$ {D}_f(t)={D}_{inc}\left(1-{e}^{- kt}\right) $$

or

3 $$ {D}_f(t)={D}_{inc\mathrm{f}}\left(1-{e}^{-{k}_{\mathrm{f}}t}\right)+{D}_{inc\mathrm{s}}\left(1-{e}^{-{k}_{\mathrm{s}}t}\right) $$

where *D*_*inc*_ was a fitting parameter describing the fraction of amide protons (%) that exchange with the rate constant *k* (min^–1^), and *D*_*incf*_, *D*_*incs*_ were the fitting parameters for two populations exchanging with the corresponding rate constants, *k*_*f*_ and *k*_*s*_, respectively. Discrimination between the mono- and bi-exponential models as well as between bi-exponential models with one of the *k* values fitted or fixed [52, 55, 56] was based on an Akaike’s Information Criterion [57] and also on a two-parameter statistical Snedecor’s *F*-test [58] for the models with different numbers of degrees of freedom.

The intrinsic rate constant values, *k*_*int*_, for the investigated peptic peptides were calculated according to the following numerical procedure: the peptide amide hydrogen exchange rates for the individual amino acids pairs were predicted by the SPHERE program [59–61]. These values were used to calculate the expected number of deuteria incorporated by each peptic peptide based on its sequence, and further to determine the exchanged fraction at nine different time points ranging from 0.001 to 60 s. The value of *k*_*int*_ for each peptide was then obtained as a fitting parameter according to Equation 2.

### Conformational Equilibrium

The Gibbs free energy of activation, *ΔG* ^*++*^, defining the height of the barrier for the transition from the closed to open RRM conformation upon the EX1 [62]. was estimated from the Eyring-Polanyi transition state theory [63] as:

4 $$ \varDelta {G}^{++}=-R\cdot T\cdot \ln \frac{k^{++}\cdot h}{k_B\cdot T} $$

where *R* - universal gas constant, *T* - absolute temperature, *h* - Planck constant, *k*_*B*_ - Boltzmann constant, and *k* ^*++*^ was the kinetic rate constant (s^-1^). The molecules undergo two concurrent processes: the reversible conformational transition and the irreversible HDX according to the exchange-accessibility provided by a particular conformation. The experimentally measured intensities of MS signals for the peptides in the closed conformation that exchange incrementally with the kinetics of the close state come only from this fraction of the closed state population that never once crossed over the conformational barrier to the open state before. Analogously, the intensities of MS data at the characteristic *m/z* range for the open conformation come not only from the actual open state population but also from these closed state molecules that underwent to the open state and could exchange immediately with the open state kinetics. Thus, the observed apparent decrease in the closed conformation population determined by HX-Express2 after subsequent time intervals of the H/D exchange, *P*_*cl*_(*t*), is related to the conformational change kinetic rate constant, *k* ^*++*^, according to the equation:

5 $$ {P}_{cl}(t)={P}_{cl}(0)\cdot \exp \left(-{k}^{++}\cdot t\right) $$

The difference in the Gibbs free energies of the closed and open conformations, *ΔG*, was calculated from the initial population, *P*_*cl*_(0), according to the equation:

6 $$ \varDelta G=-R\cdot T\cdot \ln \left(\frac{P_{cl}(0)}{1-{P}_{cl}(0)}\right) $$

The Gibbs free energy attributed to the local protein stability (minimum on the local energy landscape) upon the EX2 conditions [62] was calculated as:

7 $$ \varDelta {G}^{local}=-R\cdot T\cdot \ln (p) $$

where *p* was the protection factor obtained from the intrinsic rate constant, *k*_*int*_, and the measured rate constant, *k*, corresponding to a given amide hydrogen population, according to the equation:

8 $$ p={k}_{int}/k $$

### Fluorescence Spectroscopy

Fluorescence spectra were measured on Fluorolog 3.11 (Horiba) for tryptophan (NATA) at 22 μM in water, 1:1 water:methanol, methanol, propan-2-ol, while for SD at 2 μM and eIF4E at 2.75 μM in buffer, in a thermostatted micro-cuvette (Hellma) at 15 °C, with excitation wavelength of 280 nm. Concentrations of proteins and NATA were chosen to have equimolar amounts of tryptophan residues in each sample. Energy at maximum of the fluorescence spectra was calculated as:

9 $$ E=h\cdot c/{\lambda}_{max}, $$

where *h* was the Planck constant, *c* was the speed of light in vacuum, *λ*_*max*_ was the wavelength of the maximum.

During the tryptophan-based thermofluorescence assay, the fluorescence emission signals at 350 and 330 nm were measured every 10 s with the integration time of 4.6 s, at the excitation wavelength of 280 nm. The temperature gradient was 1 °C/min. The actual temperature was measured inside the cuvette with the thermocouple and recorded by the thermostat. The assay for SD was repeated five times at different SD concentrations, from 1 to ~13 μM. The assay for eIF4E was performed at 2.75 μM. The melting temperature was determined from a non-linear fitting of the Boltzmann sigmoidal curve:

10 $$ {F}_{350}/{F}_{330}(T)={FF}_i+\left({FF}_f-{FF}_i\right)/\left(1+\exp \left(\left({T}_m-T\right)/ slope\right)\right), $$

where *T*_*m*_ was the melting temperature in °C, *slope* was a cooperativity coefficient, *FF*_*i*_ and *FF*_*f*_ were the initial and final plateau, respectively.

### Bioinformatics

The human GW182 RRM structure prediction was done by I-TASSER [64] with default settings. The best model had a C-score of –1.38 and expected TM-score 0.54 ± 0.15. The RRM figures and contact map were prepared by Discovery Studio v3.5.0 (Accelrys Software Inc.) based on the structures aligned by Swiss-PdbViewer v4.0.4 [65]. Disorder tendency was predicted by the MetaDisorderMD2 meta-server [66] utilizing a variety of algorithms [67–82] to get a well-balanced average prediction. Peptide coverage was drawn using Draw Map [83]. Sequence alignment was performed in Align, Uniprot Clustal Omega [84]. The GW182 flexibility and normal modes were checked by CABS-Flex [85] and WEBnm@ [86] servers.

### Circular Dichroism Spectroscopy

Far-UV CD spectra were recorded with the MOS-450/AF-CD spectrometer from Bio-Logic, with a Xe lamp as a source of light, in the wavelength range of 190–260 nm, in a thermostatted 0.1 mm quartz cuvette at 20 °C. Samples of both the fresh and frozen-thawed protein at 24.4 μM in 50 mM Tris, 150 mM NaCl, 2 mM DTT, 1 mM EDTA, pH 7.0, without and with 10% glycerol, respectively, were prepared three times. The measurements for each protein and buffer sample were repeated twice, with an acquisition time of 10 s at each nanometer. The scans were averaged and the reference CD spectra of respective buffers were subtracted from the protein spectra.

## Results and Discussion

### Structural Dynamics Properties of the GW182 Silencing Domain

Homology modeling of the human GW182 RRM structure (Figure 1c) shows that this domain is similar to the *D. melanogaster* GW182 RRM except for a slight distortion of the shortest β3′-strand. The secondary structural elements corresponding to those identified in the *Dm* RRM are formed by the amino acids in the following ranges of the human protein sequence: β1: 1515-1519; α1: 1528-1537; β2: 1540-1546; β3: 1551-1556; α2: 1559-1569; β3′: 1572-1574; β4: 1578-2583; and α3: 1587-1594 (Figure 1a, c). Detailed predictions for the whole GW182 silencing domain indicate a mostly disordered character, with the exception of the RRM domain and a few short regions that exhibited a lower disorder tendency (Figure 1d). The PAM2 motif is among them, but not CIM1. However, neither the predicted presence of the well-defined human GW182 SD RRM structure nor the unfolded character of the remaining part of the structure have yet been confirmed experimentally.

**Figure 2:**
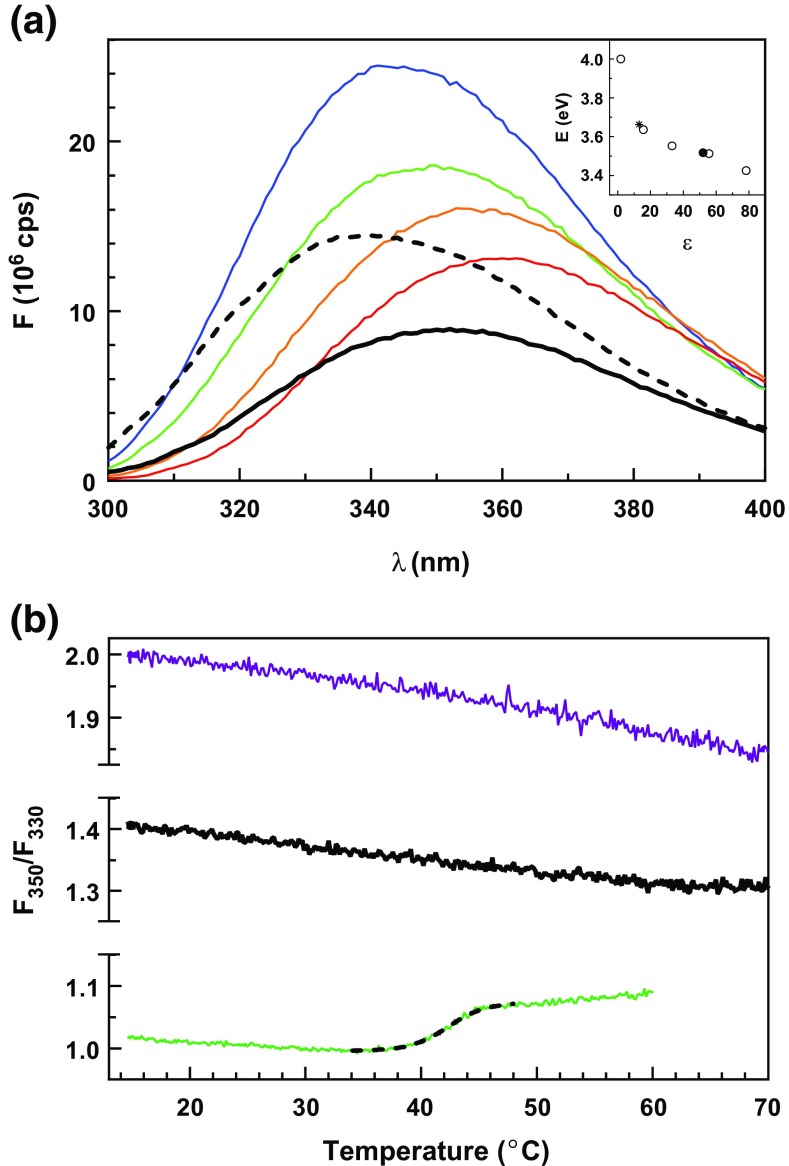
**(a)** Fluorescence spectrum of SD at 2 μM in buffer (thick black line) together with spectra of tryptophan (thin lines) (NATA) at 22 μM in water (red), 1:1 water:methanol (orange), methanol (green), propan-2-ol (blue), and eIF4E at 2.75 μM (thick broken line). Inset: dependence of the energy of the fluorescence spectra maximum on the solvent dielectric constant for tryptophan (○), SD (●), and eIF4E (*); **(b)** thermofluorescence assay for SD at 1 μM (black), NATA at 11 μM (purple), and eIF4E at 2.75 μM (green) with the Boltzmann sigmoidal melting curve (black broken line)

Some experimental indications whether GW182 silencing domain is intrinsically disordered can be inferred from UV spectroscopy, i.a. environment sensitive fluorescence measurements or circular dichroism. The SD sequence contains 11 tryptophans, 10 of which are outside the RRM domain, and one of them is localized at the border of the structured part, as predicted by I-TASSER (Figure 1a). The fluorescence spectrum of SD (Figure 2a) was compared with tryptophan in solvents with decreasing dielectric constants: water, 1:1 water:methanol, methanol, and isopropanol, as well as with eIF4E, a globular protein containing eight tryptophans, where five of them are buried and three of them are water accessible in the *apo* state. The λ_max_ of the SD emission spectrum, 352 nm, resembles that of tryptophan in the water:methanol mixture, contrary to eIF4E with the λ_max_ of 338 nm. The results can be used to semiquantitatively estimate the average dielectric constant experienced by the tryptophan indole rings of the two proteins, which proved to be ~52 for SD and ~13 for eIF4E. The comparison of the SD thermofluorescence assay with NATA as a standard for a completely water accessible tryptophan and eIF4E as a standard for a globular protein without disulfide bridges shows that for SD, contrary to eIF4E (T_m_ = 42.43 ± 0.08 °C), no unfolding transition could be detected (Figure 2b). Thus, the GW182 SD tryptophans are, by average, much more water-exposed than in eIF4E, but not totally, since some water protection is visible due to the presence of the SD protein chain.

**Figure 3.**
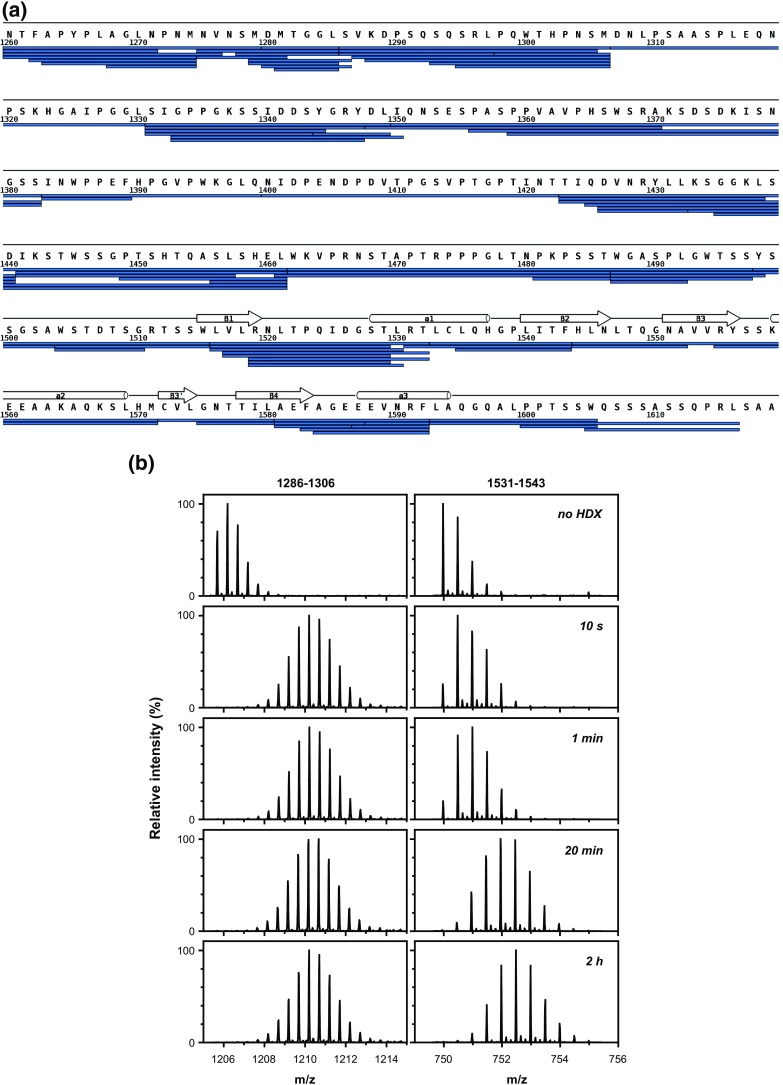
**(a)** Reproducible 99% coverage of the GW182 sequence by redundant peptides obtained by pepsin digestion; **(b)** representative mass spectra of a peptic GW182 peptide with immediately exchanged amide protons (left panels, residues 1286-1306, charge +2) encompassing the CIM1 motif and a peptide with slowly exchanging amide protons (right panels, residues1531-1543, charge +2) belonging to the most buried part of the RRM domain, at consecutive HDX time points

In order to address the issue of structural dynamics of the GW182 silencing domain in a more precise manner, we performed hydrogen–deuterium exchange experiments for different deuteration times. The reproducible pepsin digestion pattern covering 99% of the human GW182 SD sequence is shown in Figure 3a. The number of pepsin-generated peptides and the sequence coverage were similar for both fresh and frozen protein samples. This complete sequence coverage with a rich redundancy enabled us to conduct a systematic analysis of the susceptibility for the H/D exchange of particular protein regions. The digestion pattern by pepsin indicated preference for cleavage within the α-helices or β-strands of the RRM domain and not at loops, even though the pepsin buffer contained guanidinium hydrochloride at 0.67 M. A similar preference for the peptide bonds within the defined secondary structural elements rather than in unfolded regions was reported earlier for pepsin digestion of the eIF4E protein [52] and for neutrophil elastase digestion of proteins containing RRM domains [87].

The protection against the exchange reflects the stability of the intramolecular hydrogen bonding network and thus describes the protein structural dynamics on a broad time scale, related to the frequency of transient breakages of particular hydrogen bonds and their accessibility to solvent [88]. A comparison of representative mass spectra of selected peptic GW182 SD peptides after different period of the H/D exchange are shown in Figure 3b.

**Figure 4:**
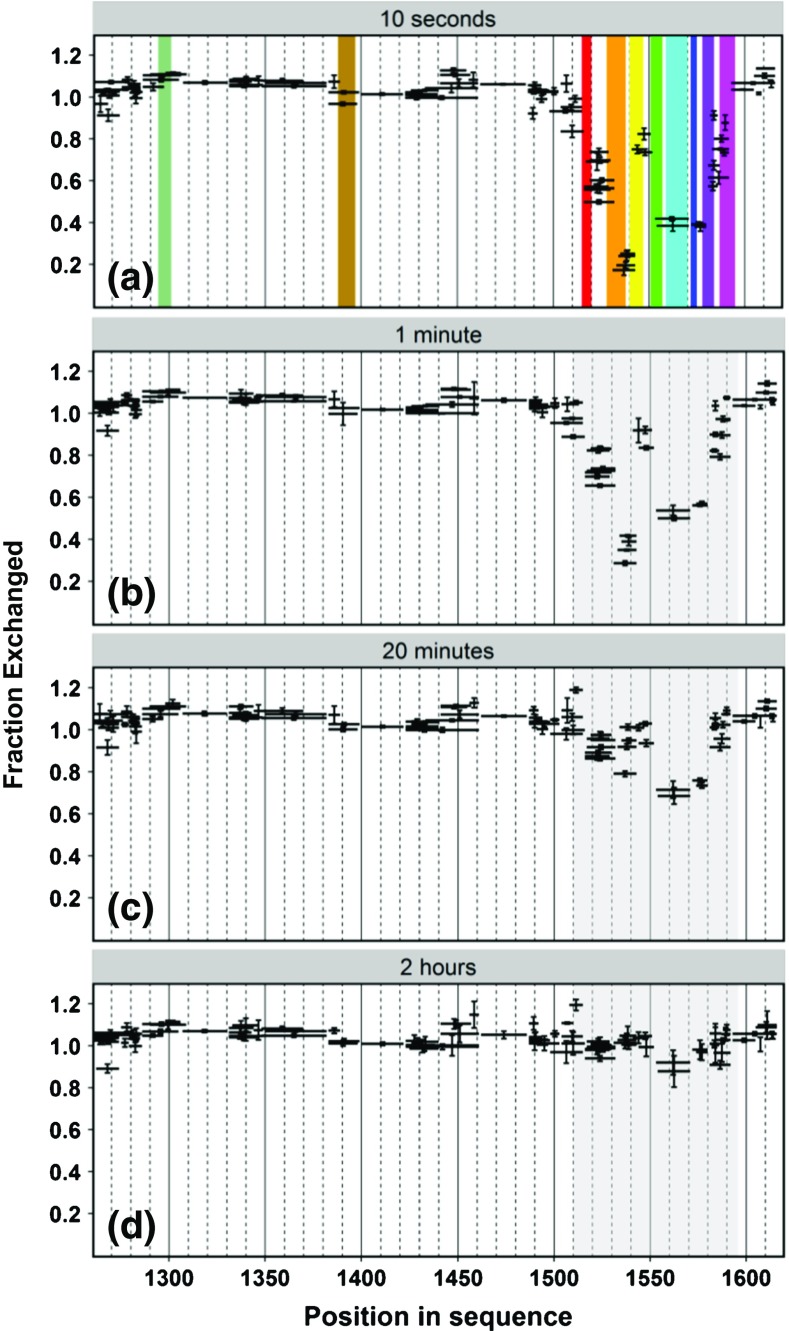
**(a)** Fraction of the exchanged population of amide hydrogens (with vertical standard errors) obtained for pepsin-generated GW182 SD peptides marked as horizontal black bars, after 10 s of deuteration, corrected for back-exchange of the individual peptides, overlayed with secondary structural elements assigned and colored as in Figure 1a, c; **(b)** the same as in **(a)** after 1 min, **(c)** after 20 min, and **(d)** after 120 minof deuteration

The majority of the identified peptides displayed the complete exchange of all exchangeable amide protons even at the shortest exchange time (10 s, Figure 4a) in the same manner as for the peptide encompassing the CIM1 motif (residues 1286-1306), shown in the left panels of Figure 3b. The results obtained for all peptides analyzed along the GW182 SD sequence at consecutive HDX time points (10 s, 1 min, 20 min, 2 h) are summarized in Figure 4a–d. These results provide the experimental evidence for the following conclusions: (1) CIM1 reveals no detectable secondary structure; and (2) most of the GW182 silencing domain sequence is not engaged in intramolecular hydrogen bonding, which would result in protection against HDX.

Contrary to the unstructured part, a GW182 SD region encompassing the amino acids 1504-1592 clearly displays substantial protection, prominent at the first two time points. This can be associated with the presence of an ordered α/β protein structure, since this region corresponds to the RRM domain of the protein. The right panels of Figure 3b present the spectra of the initially most protected peptide (1531-1543) belonging to the RRM domain (Figures 1b, c and 3a). All the RRM peptides were gradually exchanged upon time, although the details of their exchange kinetics differed significantly. The HDX pattern reflecting the protein structure is much less visible after 20 min, and completely disappears after 2 h of deuteration, except for the peptides encompassing the α2 helix that displayed a residual protection. This means that the overall protection of the RRM residues against the H/D exchange is low, which indicates significant flexibility and remarkable structural dynamics throughout the whole RRM domain.

The HDX results (Figure 4a) indicate also that the secondary structural assignments modelled by I-TASSER (Figure 1a, c) should be extended more towards the N-terminus of the protein, and include the residues down to Arg1511, involved probably in the β1 strand, since four peptides within this region (1498-1515, 1504-1515, 1504-1516, and 1508-1515, except for 1504-1510) display some residual protection against the exchange at 10 s.

### Kinetic Parameters of the GW182 RRM Hydrogen–Deuterium Exchange

**Figure 5:**
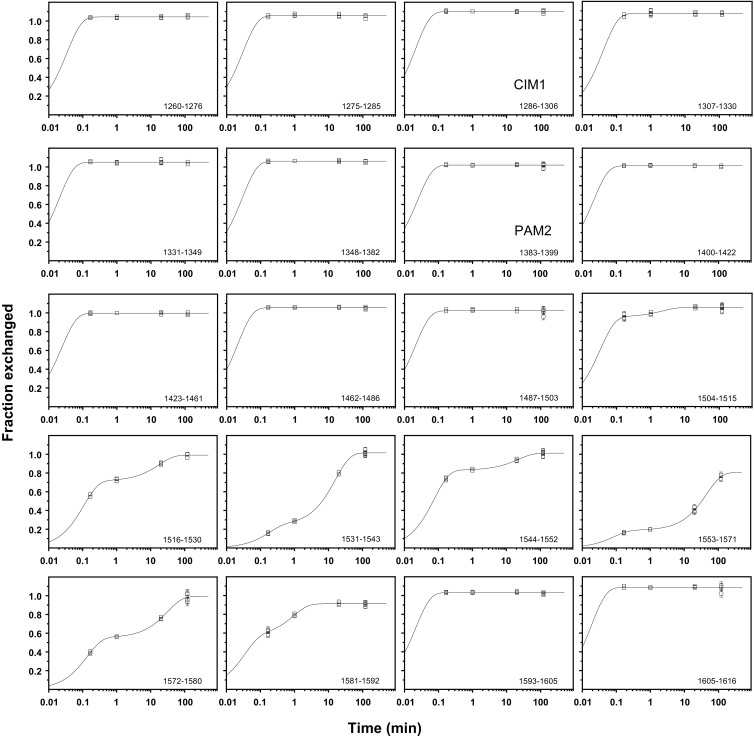
Kinetic plots of hydrogen-deuterium exchange for non-redundant pepsin-generated peptides of GW182 SD; data points represent the fraction of the amide hydrogen population exchanged to deuteria after consecutive time intervals of HDX; experimental uncertainty of the individual data points are shown as vertical bars; mono- or bi-exponential functions were fitted to the experimental data points according to Equation 2 or Equation 3

Kinetic plots for the representative peptic peptides covering the whole GW182 silencing domain sequence are shown in Figure 5. Detailed analysis of the bi-exponential deuterium incorporation kinetics for the individual peptides revealed that each RRM peptide is characterized by two populations of amide hydrogens, referred to as fast and slow.

The fast populations, *D*_*incf*_, incorporate deuteria with the *k*_*f*_ rate constants in the range of ca. 6 to 30 min^–1^, whereas the slow populations, *D*_*incs*_, are much more protected and the exchange occurs with *k*_*s*_ in the range of ca. 0.02 to 2 min^–1^. The detailed kinetic parameters for seven peptides encompassing the RRM domain are gathered in Table 1. Each peptide is attributed to its secondary structural element marked in Figures 1 and 4. The percentage of the slow population, *D*_*incs*_, shows that the most protected peptides correspond to the α1/turn/β2 and β3/turn/α2/turn fragments, while 20% of the latter (residues 1553-1571) remains unexchanged even after 2 h.

**Table 1:** Kinetic Parameters of Hydrogen–Deuterium Exchange for Pepsin-Generated Peptides of the GW182 RRM Domain. The Percentage of the Fast and Slow Amide Hydrogen Populations, *D*_*incf*_ and *D*_*incs*_, that Incorporate Deuterium with the *k*_*f*_ and *k*_*s*_ Rate Constants, Respectively; the Site-Specific Intrinsic Rate Constants, *k*_*int*_; and the Gibbs Free Energies Related to the Local Stability in the EX2 Conditions, ΔG_f_^local^ and ΔG_s_^local^

GW182	fold	D_incf_ (%)	k_f_ (min^–1^)	D_incs_ (%)	k_s_ (min^–1^)	k_int_ (min^–1^)	ΔG_f_^local^ (kJ⋅mol^–1^)	ΔG_s_^local^ (kJ⋅mol^–1^)
1504-1515	coil/β1	94.8 ± 1.6	30 ^a^	10.3 ± 1.7	0.5 ± 0.3	801	–8.0	-18.0
1516-1530	β1/turn/α1	71.5 ± 1.0	9.3 ± 0.4	27.7 ± 1.2	0.056 ± 0.006	358	–8.9	-21.3
1531-1543	α1/turn/β2	24.1 ± 1.1	5.8 ± 0.8	77.4 ± 1.4	0.062 ± 0.002	426	-10.5	-21.5
1544-1552	β2/turn/β3	83.0 ± 1.1	13.1 ± 0.9	18.3 ± 1.4	0.045 ± 0.009	734	-9.8	-23.6
1553-1571	β3/turn/α2/turn	18.7 ± 1.1	12 ± 3	62 ± 2	0.022 ± 0.002	540	-9.3	-24.6
1572-1580	β3’/turn/β4	55.2 ± 1.6	7.4 ± 0.7	44 ± 2	0.032 ± 0.005	403	-9.7	-23.0
1581-1592	β4/turn/α3	55.9 ± 1.8	30 ^a^	35.3 ± 1.9	1.08 ± 0.13	336	-5.9	-14.0
*after freezing-thawing cycle: double conformation of 1554-1571 peptide (α2) in the EX1 conditions*								
*closed*	β3/turn/α2/turn	15.1 ± 1.9	30 ^a^	28 ± 3	0.1 ^a^			
*open*	β3/turn/α2/turn	89.8 ± 1.0	10.4 ± 0.4	5.9 ± 1.2	0.1 ^a^			

^**a**^Fixed values, according to [52, 55, 56]

The local structural dynamics of the protein (without the freezing-thawing cycle) can be analyzed within the EX2 conditions in terms of the Gibbs free energies, ΔG_f_^local^ and ΔG_s_^local^, related to the local stability (minimum on the local energy landscape) of peptide fragments containing fast and slowly exchanging protons. These values were calculated from the protection factors based on the site-specific intrinsic rate constants, *k*_*int*_, for given amino acid sequences. The lowest *k*_*int*_ value of 300 min^–1^ was obtained for the PAM2 containing peptide, whereas the average value for the N- and C-terminal GW182 SD parts, flanking the RRM domain was 630 min^–1^. The most negative ΔG_s_^local^ value of about –25 kJ/mol was obtained under the native conditions for the α2 helix containing peptide.

### Dual Conformation of the RRM α2 Helix

In general, the GW182 RRM HDX pattern resembles the shape of the predicted disorder probability profile for the peptides within the RRM domain (Figure 1d). However, two significant divergences are clearly visible after closer inspection, i.e., (1) the fragment predicted to be the best ordered (residues 1540-1550) is relatively the most susceptible to exchange among the peptic RRM peptides (Figure 4a, yellow stripe), and (2) the higher relative local disorder tendency predicted by multiple algorithms for the 1555-1570 region corresponding to the α2 helix is in apparent disagreement with HDX, which shows that this RRM fragment is most protected (Figure 4d). While the first observation can be explained by the fact that this fragment corresponds to the β2 strand together with the subsequent tight turn (Figure 1a, c) that both can be concurrently well ordered and partially solvent- and exchange-accessible, the latter contradiction seemed to be highly puzzling, especially since the inspection of all available unliganded RRMs in the PDB showed that just this α2 helix is the most rigid and precisely structurally defined fragment (with the lowest RMSD values) in the RRM structures resolved by NMR. We checked the low-frequency normal modes and the local flexibility, modeled on known PDB templates. The analyses confirmed that the α2 helix residues should not undergo large amplitude fluctuations (Supplementary Figure 1). Bearing in mind that RRM domains are observed to be unstable at higher concentrations upon longer times, we checked how the α2 helix conformation could be affected by the freezing-thawing cycle.

**Figure 6.**
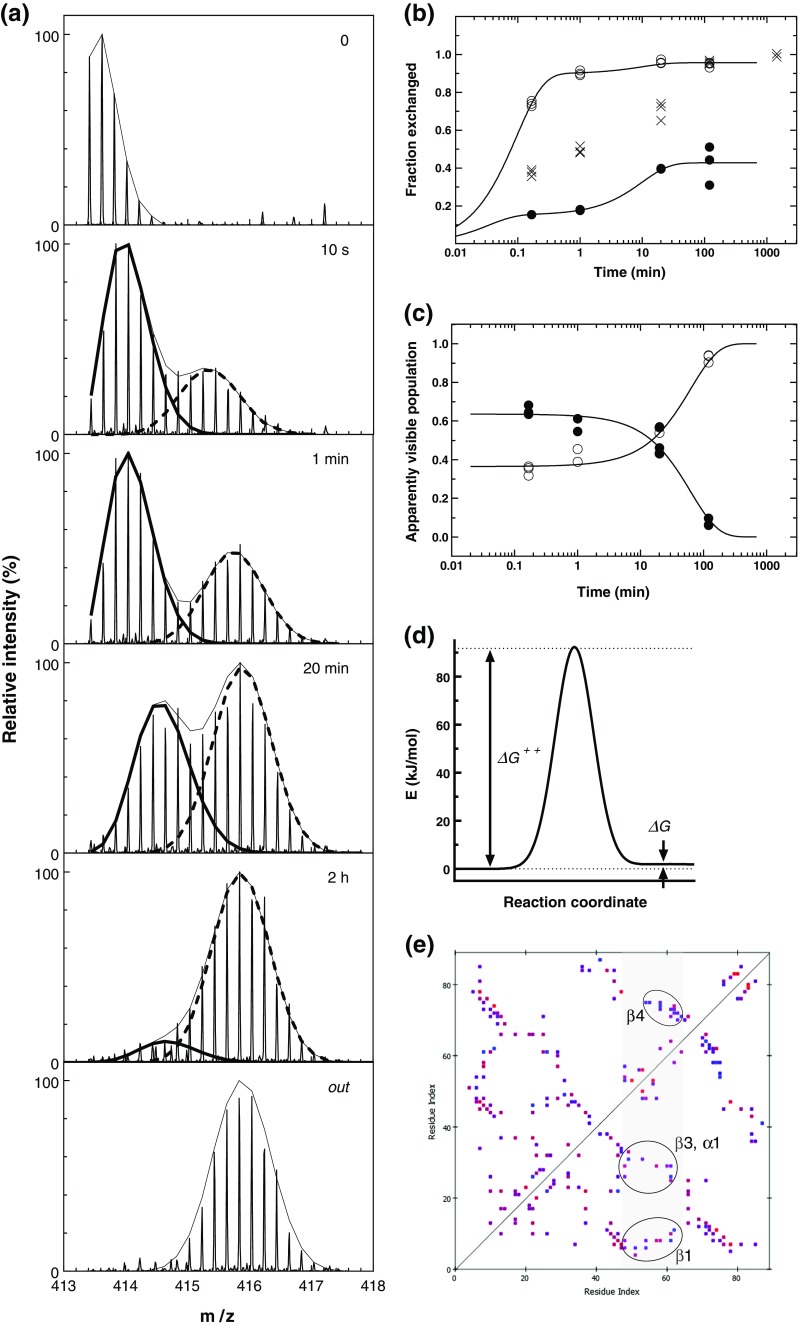
**(a)** Bimodal isotopic envelopes for the GW182 SD RRM α2 peptide (residues 1544-1571, charge +5) after given time of H/D exchange; (—) closed conformation, (- - -) open conformation; **(b)** Kinetic plots of HDX for the 1544-1571 peptide; data points represent the fraction of the amide hydrogen population exchanged to deuteria after consecutive time intervals of HDX for (●) closed α2 peptide conformation, (○) open conformation, (x) average values; **(c)** apparent populations in the closed and open state as seen by HDX: (●) the fraction of the population of the closed state molecules that never once underwent the change to the open conformation, (○) population of the open state together with the fraction of the population of the closed state molecules that at least once underwent the change to the open conformation; **(d)** Gibbs free energy barrier for the conformational equilibrium of the α2 peptide; **(e)** contact map (within 3.5 Å) for the side chains of the GW182 SD RRM (residues 1509-1595). The α-helix 2 region is marked as gray band. The contacts between the α2 residues with the β1, β3, β4, and α1 are encircled

The mass spectra recorded for the frozen-thawed samples revealed clearly that the peptides encompassing the α2 helix (residues 1544-1571) displayed a bimodal isotopic envelope (Figure 6a). The shortest of them was RYSSKEEAAKAQKSLHM (1555-1571, Figure 7a). This means that the α2 helix exists in two distinct conformations. The more exchange-protected conformation of this peptide, denoted to as the closed state, has a similar H/D exchange kinetics (Figure 6b) as the sole form detected for the nonfrozen samples (Figure 5). The more exchange-competent conformation, denoted to as the open state, displays full deuteration after 20 min of the exchange (Figure 6a, b). The populations of the two states at the zero exchange time are comparable (64% versus 36%, Figure 6c), which corresponds to the difference in the Gibbs free energies between these two states is less than *RT*, ΔG = 1.4 kJ/mol (Figure 6d).

**Figure 7:**
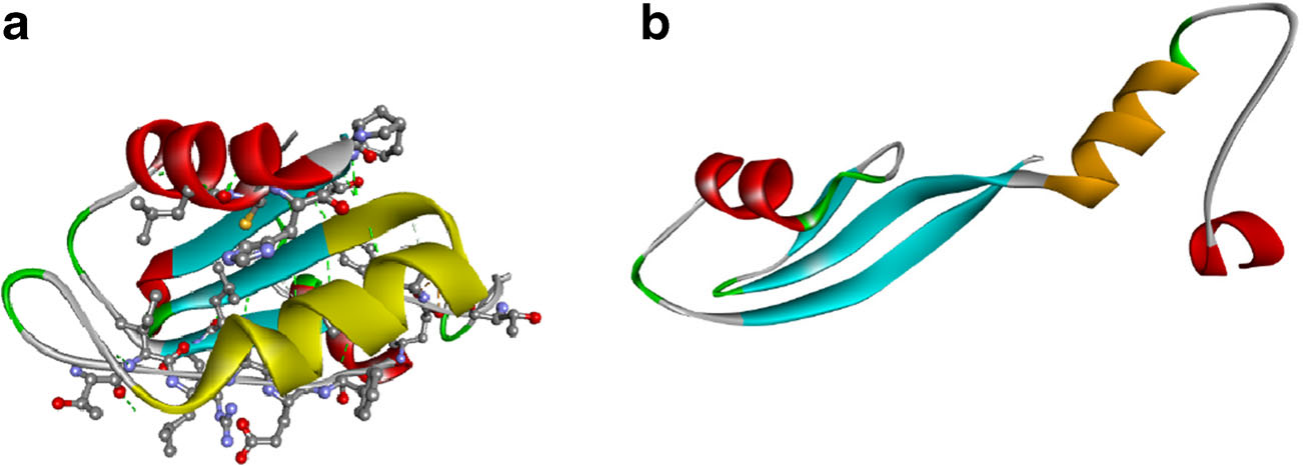
**(a)** Human GW182 SD RRM model with the shortest α2 peptide (RYSSKEEAAKAQSLHM) that displays the bimodal isotopic envelope, marked yellow. Residues in non-covalent contacts with α2 (≤3.5 Å, excluding internal α2 contacts) belonging to α1, β1, β3, β4 (T15, S17, W18, L19, L21, R22, L36, C37, H40, P42, I44, T45, TILAEF(81-86), E89) shown as CPK balls-and-sticks; **(b)** crystal structure of a single RRM domain truncated from human PARN, with the α2 helix that is swapped in the RRM dimer (PDB id code: 3CTR [89]) marked gold

The observed type of bimodal distribution is characteristic for the EX1 kinetics, where the chemical H/D exchange is faster compared with a characteristic time constant for global conformational changes or subunit dissociation that break most of the solvent-exposed hydrogen bonds, before refolding or rebinding takes place [4, 62, 90]. The equilibrium conformation populations remain constant at given temperature, but an apparently observed decrease in the population of the α2 peptide in the closed conformation and the corresponding apparently observed increase in the open conformation population are visible (Figure 6c) due to the irreversible exchange in excess of D_2_O during the time spent by the peptide in the open state before coming back to the closed state (see Materials and Methods). This makes it possible to estimate the kinetic rate constant, *k* ^*++*^, for the transition over the barrier from the closed to open conformation within the Eyring-Polanyi transition state theory. Since the observed process is very slow, with *k* ^*++*^ in the order of 1 h–^1^, the energy barrier that separates the conformations is very high, about 90 kJ/mol (Figure 6d), which is ~36-fold higher than *RT*. An average binding free energy contribution from a single non-covalent contact in a water-accessible protein region ranges from –3 to –8 kJ/mol and is similar for salt bridges, hydrogen bonds, van der Waals interactions, and hydrophobic contacts due to entropic effects related to the presence of counter-ions, surrounding water molecules in buffer and to the penalty of ordering [91]. This means that the transition from the closed to open conformation would require a transient break of more than a dozen structure stabilizing non-covalent contacts within the relatively small RRM domain. Figures 5e and 6a presents the non-covalent side chain contacts formed by the α2 helix with the residues within the distance of 3.5 Å belonging to the other secondary structural elements: β1, β3, α1, and β4 strands. The peptides of the frozen-thawed protein containing the T1512, S1514, W1515, L1516, L1518, R1519, L1533, C1534, H1537, P1539, I1541, T1542, TILAEF(1578-1583), and E1586 residues (Figure 7a) displayed also dual isotopic envelopes but to a lesser extent.

An example of a rupture of such a great number of contacts leading to a different but energetically equivalent conformational state for a single RRM domain in the absence of a larger globular protein context can be found in the PDB, i.e., the RRM of the poly(A)-specific ribonuclease (PARN) [89]. Full length PARN is a homodimer composed of three domains in each subunit (the nuclease, R3H, and RRM) [19, 92], and its RRM domain is prone to dimerization [93]. When crystallized alone, the truncated RRM domain of PARN forms a dimer with a perfect swapping of just the α2 helices between the RRM molecules [89] (Figure 7b). Both a putative transition of the α2 helix as a whole to a swapped conformation, and a cooperative movement with a transient unfolding of intra-helical hydrogen bonds, could yield similar EX1 characteristics. However, it seems hardly possible that the Gibbs free energy of unfolded conformations of the α2 helix, i.e., the most tightly folded RRM part (according to the PDB NMR data) could be almost equal to those of the helical state (Figure 6d).

Moreover, slow motions related to conformational changes or folding of individual protein residues are in the microsecond to millisecond timescale [94, 95], whereas characteristic times of primary nucleation phase upon protein aggregation are hours [96]. The double isotopic envelope effect related to the presence of two long-lasting conformations observed for the frozen-thawed samples could be thus rather a result of a conformational rearrangement of the whole α2 helix. The possibility of the existence of α2 in two conformations could, in turn, explain why the disorder tendency prediction (Figure 1d) is higher for the tight α2 helix than for the surrounding area.

A question arises whether – alternatively – the GW182 RRM region that displays the bimodal isotope envelopes might serve as a nucleation region for protein aggregation during the freezing-thawing cycle, and thus, if the observed EX1 kinetics of conformational changes might reflect a process of associated retarded re-solubilization. However, if this were the case, the sole conformation detected for the soluble, nonfrozen protein would correspond to the open conformation, whereas the opposite is true. The H/D exchange kinetics (residues 1553-1571 in Figure 5) detected for fresh samples is similar to the closed conformation of the 1554-1571 peptide (Figure 6b) in the frozen samples (for kinetic parameters, see Table 1).

**Figure 8:**
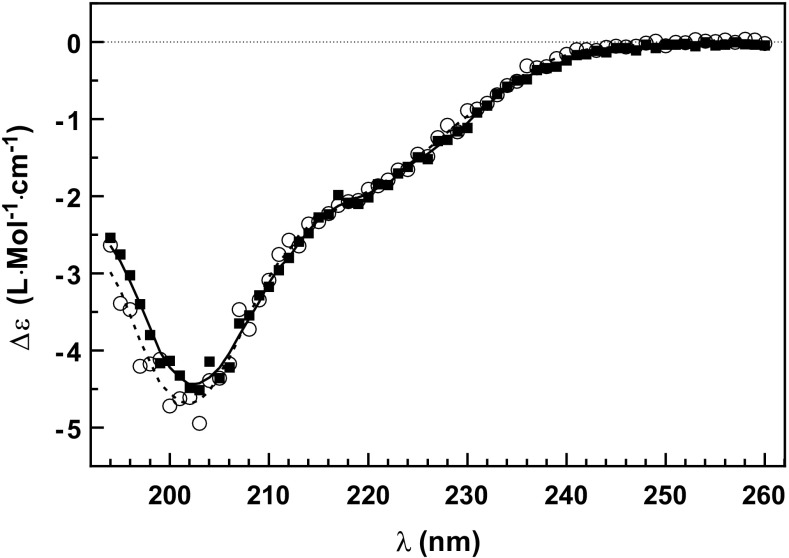
Far-UV CD spectra of GW182 silencing domain; (○) nonfrozen protein, (■) protein after the freezing-thawing cycle

To further check whether the bimodal isotope envelopes in HDX MS could be evidence of conformational changes at the secondary structure level that could lead to protein aggregation, we measured the far-UV CD spectra of GW182 SD (Figure 8). The main negative peak at 202 nm and a shoulder at 220 nm point to the prevailing contribution coming from disordered residues with much lesser contributions of the residues in the α-helical, antiparallel β-sheet, and β-turn conformations. The spectra of both the freshly prepared and frozen-thawed protein are very similar. The small difference observed in the range of the shortest wavelengths (<200 nm) might be attributed to a decrease in β-turn or coil content, but the putative change is in the order of the experimental uncertainty. This means that the populations of the amino acid residues in varying secondary structure conformations present in the fresh and frozen samples are essentially unchanged, and the putative slight decrease in β-turn would be in agreement with the movement of the whole α2 helix (Figure 7). We can thus conclude again that the dual isotopic envelopes in the HDX MS of the frozen GW182 samples are related to the conformational change at the RRM tertiary structure level rather than to direct aggregation or misfolding. The exact numerical determination of the secondary structure contents by means of available CD software failed due to the mainly unstructured character of the protein, while the software are focused and trained on protein sets with known crystal structures.

Detailed analyses of the molecular properties of proteins containing intrinsically disordered regions are still a challenging task, where HDX MS appears to be the method of choice. Conventional approaches that address protein structure-related issues based on NMR and crystallography require high concentrations of proteins. Not all proteins can be produced in such quantities, while many aggregate or precipitate. HDX MS is a far less protein- and time-consuming approach than multidimensional NMR. Moreover, IDPs do not exhibit a stable 3D conformation in physiological conditions, and thus are not suitable for crystallography. HDX MS can provide an insight into the structural dynamics and intermolecular interactions of such proteins, since this method detects local stability of the hydrogen-bonding network, which reflects protein flexibility, conformational transitions, and – indirectly – solvent accessibility, at a peptide level. This is a significantly higher resolution compared with spectroscopic techniques such as circular dichroism or steady-state fluorescence and correlation spectroscopy that give structurally averaged data. Even for globular domains like the RRM, HDX MS is advantageous in studying the long time-scale conformational changes, which would be hardly observable by other methods, including mutation-based labeling for FRET or DEER ESR. HDX MS can detect conformational inhomogeneity based on bimodal isotope envelopes from 1-d measurements, directly after protein purification. Our results show that the influence of the environmental conditions on the RRM domain structure should not be neglected, since this may have an impact on interactions with other molecules.

Since GW182 SD is a mainly intrinsically disordered protein and is available in limited amounts, HDX-MS was deemed to be the most suitable method for studying its structural dynamics in solution. The results described here form a crucial biophysical basis for further experiments that address the nature of molecular interactions of GW182 SD with the CNOT1 subunit of the CCR4-NOT de-adenylation machinery in the process of miRNA-mediated gene silencing.

## Conclusions

Our work reveals the hydrogen–deuterium exchange mass spectrometric description of an RNA Recognition Motif, which is one of the most frequently occurring canonical protein domains. We show that the RRM structure reveals the alternating HDX pattern, composed of the regions containing amide hydrogens that are more or less exchange-competent, even when the RRM domain is studied in the context of a larger fragment of the generally disordered protein, which is not suitable for NMR due to its size and aggregation at higher concentrations. The experimentally determined structural dynamics of the GW182 SD RRM correlates well, in general, with the predicted α/β content. We found that the RRM structure is highly dynamic, since the complete H/D exchange is achieved in 2 h. These results indicate that water molecules can penetrate the whole domain. The HDX studies suggest also that the α2 helix of the RRM can change its conformation after a freezing-thawing cycle, which might be a clue for explanation of a possible RRM aggregation pathway. These studies have provided the experimental proof that the silencing domain (SD) of GW182, except the RRM domain, is indeed natively unstructured. In particular, the CCR4-NOT interacting motif 1 does not display any evidence of a secondary structure in the HDX MS timescale.

## Electronic supplementary material

Supplementary Figure 1 (DOCX 39 kb)
