# Supplementary material for: Structural Dynamics of the GW182 Silencing Domain Including its RNA Recognition motif (RRM) Revealed by Hydrogen-Deuterium Exchange Mass Spectrometry
Source: J Am Soc Mass Spectrom. 2017 Oct 27;29(1):158–73. doi: 10.1007/s13361-017-1830-9 (PMC5785596; doi:10.1007/s13361-017-1830-9)

**Supplementary Figure 1**

The RRM flexibility profile. The numbering corresponds to the GW182 RRM domain alone. The shortest peptide that contains the tight α2 helix and displays the bimodal isotopic envelope encompasses here the residues no. 59-74.


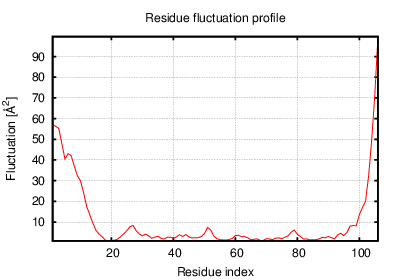

Supplement: Supplementary file 1 — (DOCX 39 kb) [file 13361_2017_1830_MOESM1_ESM.docx]
